# *Platyhypnidium aquaticum* as Bioindicator of Metal and Metalloid Contamination of River Water in a Neotropical Mountain City

**DOI:** 10.3390/plants9080974

**Published:** 2020-07-31

**Authors:** Ángel Benítez, Samuel Torres, Ramiro Morocho, Washington Carrillo, David A. Donoso, James Calva

**Affiliations:** 1 BIETROP, Departamento de Ciencias Biológicas, Universidad Técnica Particular de Loja, San Cayetano s/n, 1101608 Loja, Ecuador; torressam1030@gmail.com (S.T.); jrmorocho@utpl.edu.ec (R.M.); 2 Maestría en Biología de la Conservación y Ecología Tropical, Universidad Técnica Particular de Loja, San Cayetano s/n, 1101608 Loja, Ecuador; wacarrillo1@utpl.edu.ec; 3 Departamento de Biología, Escuela Politécnica Nacional, Ladrón de Guevara, E11 253 Quito, Ecuador; david.donosov@epn.edu.ec; 4 Centro de Investigación de la Biodiversidad y Cambio Climático, Universidad Tecnológica Indoamérica, EC170103 Quito, Ecuador; 5 Departamento de Química y Ciencias Exactas, Universidad Técnica Particular de Loja (UTPL), San Cayetano s/n, 1101608 Loja, Ecuador; jwcalva@utpl.edu.ec

**Keywords:** bryophytes, mosses, Ecuador, passive biomonitoring, water quality

## Abstract

Water contamination is a major environmental problem in many cities of the world. Most water contamination results from industry and human activities that generate toxic substances (e.g., metals). Rheophilic and aquatic mosses are found in lotic ecosystems, and their morphological and physiological traits are responsive to ecological and pollution gradients. Here we hypothesized that the native rheophilic moss *Platyhypnidium aquaticum* (A. Jaeger) M. Fleisch exposed to polluted waters can bioaccumulate greater amounts of metals, and a metalloid, than *P. aquaticum* exposed to pollution-free water. To this aim, we tested the bioindicator capacity of the aquatic *P. aquaticum* for 15 metals (Cd, Pb, Zn, Fe, K, Ca, Na, Mn, V, Co, Ba, Cr, Al, Sr, and Mg) and one metalloid (As), in twelve river samples coming from three urban and one control zone along the Zamora river in the city of Loja. When compared to the control, our results showed that *P. aquaticum* in the Southern, Central, and Northern zones of the city bioaccumulated higher concentrations of Ba, Cd, Co, Fe, Mg, Mn, Na, Sr, Zn, and the metalloid As. On the other hand, concentrations of Al, Ca, Cr, Pb, and V in *P. aquaticum* tended to be lower in the control zone, but these differences were not significant. We suggest that the presence of these contaminants may be related to water pollution (e.g., residual discharges and a lack of treatment systems) along urban zones of the river. We report for the first time the utility of *P. aquaticum* as a model species for development of long-term biomonitoring programs of water contamination in South America. Passive biomonitoring with *P. aquaticum* can be a simple and low-cost method to obtain reliable data of the current state of water contamination with metals and metalloids in tropical regions.

## 1. Introduction

Global water supplies are increasingly contaminated with toxic elements [1,2]. In particular, water contamination with metals is a great concern, given the difficulties associated with water purification, the persistence of high toxicity levels in the environment, and the tendency of metals to bioaccumulate in consumer organisms [3,4]. Thus, environmental pollution (e.g., river pollution) is a major environmental issue affecting freshwater habitats and consequently human health [5,6,7]. In Ecuador, water contamination is of special interest given the high rates of urbanization and city industrialization and the low levels of urban planning experimented by the cities in the last decades [7,8]. City development in the country has generated large amounts of residual water and solid waste from the normalization of garbage dumps and extraction of aggregates in riverbeds [7]. There is an urgent need for sensitive and cost-effective methods to biomonitor water pollution that can aid restoration efforts across the country.

Rheophilic and aquatic bryophytes are an important part of the urban landscape [7]. They are considered as ecological indicators of water quality in urban rivers, given their capacity to bioaccumulate metals and other nutrients or contaminants found occasionally in river waters [7,9,10,11,12,13,14]. They show key characteristics that set them apart from other bioindicators: (A) many species are widely distributed, (B) contaminant absorption of chemicals is passive, and not significantly affected by biotic factors, (C) they are resistant to high levels of water contamination, (D) they are rarely invasive, and E) they can bioaccumulate metals above their physiological needs due to the absence of a cuticle in their tissues [10]. Certain aquatic bryophyte species have been widely used in recent years as bioindicators [5,15,16,17,18,19]. For instance, *Fontinalis* and *Platyhypnidium* bryophytes are the best-studied genera in terms of passive biomonitoring of metals in water, with observations from several regions in the world with chronic exposures or long-term effects of contaminants [10,20,21,22,23]. Research usually showed higher concentrations of metals in the urban zones, related to wastewater and other contaminants [10,19,21,24,25]. Bryophytes therefore constitute a simple, reliable, and low-cost tool for biomonitoring [6,10].

In the neotropics, there is little information about moss bioaccumulative properties of metals related with water pollution, with only one study (out of 73 worldwide) in Latin America [10]. In Ecuador, passive biomonitoring of water pollution with thallose liverwort has been reported only once [7]. The authors found a greater concentration of some metals in *Marchantia polymorpha* L. in urban zones of Zamora river. However, *M. polymorpha* is sometimes exposed to air, and thus it can bioaccumulate air pollutants in addition to water pollutants. To solve this issue, it is recommended to use rheophile mosses that spend all the time under water. The species studied here, *Platyhypnidium aquaticum* (A. Jaeger) M. Fleisch, is such a species. We therefore determined for the first time the bioaccumulation of 15 metals, aluminum (Al), barium (Ba), cadmium (Cd), calcium (Ca), cobalt (Co), chromium (Cr), iron (Fe), lead (Pb), magnesium (Mg), manganese (Mn), potassium (K), sodium (Na), strontium (Sr), vanadium (V), zinc (Zn), and the metalloid arsenic (As) by *P. aquaticum,* in twelve river samples coming from three urban and one control zone along the Zamora river, in the city of Loja, southern Ecuador. Our main hypothesis was that the native aquatic *P. aquaticum* bioaccumulates greater amounts of metals, and a metalloid, when exposed to contaminated waters than when exposed to waters free from pollution. By doing so, passive biomonitoring using bryophytes might be an effective technique, allowing us to evaluate simultaneously the presence of a great number of organic and inorganic compounds.

## 2. Results

The bioaccumulation of metals was high in *Platyhypnidium aquaticum* samples coming from the South, Center and North zones of the city, when compared to the control zone. The metals with the highest value in the urbanized areas were Cd, Fe, Mg, Na and Zn, and the metalloid As (Table 1). The analysis of variance revealed that the concentration of the metals Ba, Cd, Co, Sr, Fe, Mg, Mn, Na, Zn, and As (a metalloid) was different among the city zones (Table 1).

**Figure 1:**
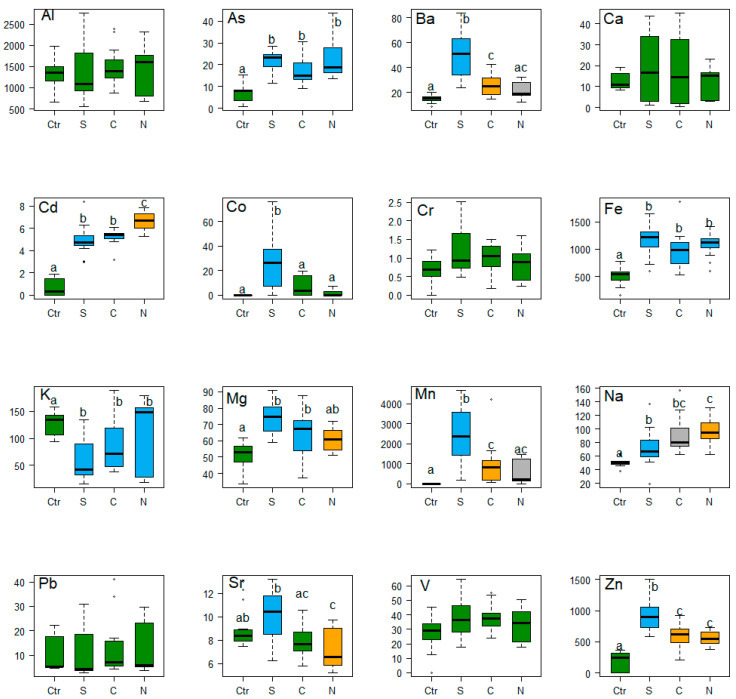
Boxplot and post-hoc Tukey (Shapiro Wilk and Bartlett’s test, *p*-valor > 0.05) and Dunn test (Shapiro Wilk and Bartlett’s test, *p*-valor < 0.05) metals concentration of aluminum (Al), barium (Ba), cadmium (Cd), calcium (Ca), cobalt (Co), chromium (Cr), iron (Fe), lead (Pb), magnesium (Mg), manganese (Mn), potassium (K), sodium (Na), strontium (Sr), vanadium (V), zinc (Zn), and the metalloid arsenic (As) in *Platyhypnidium aquaticum* (µg g^−1^). Different letters and colors indicate significant differences in metals concentration between zones. Ctr = Control, S = South, C = Center, and N = North.

The post-hoc (Tukey or Dunn) test showed that the metals Cd, Fe, Mg, Na, Zn, and the metalloid As presented higher concentrations in the South, Central and North zones, compared to the control zone (Figure 1). Manganese, cobalt, barium, and strontium showed greater bioaccumulation in the South and Central zones, compared to the Control and North zones (Figure 1). Potassium presented a different pattern compared to the rest of the metals because its highest concentrations were recorded in the Control and North zones, (Figure 1). Al, Ca, Cr, Pb, and V did not show significant differences, their concentrations being similar in almost all sampling areas (Figure 1).

## 3. Discussion

Our results indicate that, in samples of *Platyhypnidium aquaticum,* the majority of measured metals (Ba, Cd, Co, Fe, Mg, Mn, Na, Sr, Zn, and the metalloid As), showed higher levels in the South, Central, and North zones compared to control sites. These results add to a growing body of literature finding that mosses are important bioindicators of water contamination [18,19,23,26]. The sources of contamination are likely direct discharges of household waste, agricultural, and industrial activities [5,6]. Another interpretation is that these zones have a lower percentage of vegetation cover as a result of higher urbanization. For example, Cesa et al. [27] show that the concentration of such metals (e.g., Ca, Co, and Mn) gradually increases with urbanization and lower degrees of vegetation cover.

Our study is in accordance with Kosior et al. [19], who found that the concentrations of Al, Ba, Cr, Cd, Co, Fe, Mn, Pb, Sr, V, Zn, and the metalloid As typical for *P. riparioides* from unpolluted parts of the Nysa Łuzycka river of Poland were similar to those of *P. aquaticum* from the Control zone in the Zamora River. Conversely, concentrations of these pollutants were much higher for samples from urban zones of the Zamora River. Thus, our results emphasize the high levels of contamination of the examined river, especially with Ba, Cd, Co, Fe, Mg, Mn, Na, Sr, Zn, and the metalloid As. Similarly, Vasquez et al. [7] showed that the liverwort *Marchantia polymorpha* exhibited higher concentrations of Cd, Fe, and Zn in urban zones of the Zamora River when compared with controls. However, when compared with *P. aquaticum*, *M. polymorpha* bioaccumulated very low concentrations of Al (7.82 µg g^−1^), Cd (0.01), Fe (0.34), Mn (0.23), and Zn (3.08), and the metalloid As (0).

On the other hand, K concentration was highest in the control zone in comparison with the low concentrations of urbanized zones. These results may be explained by agricultural and grazing activities in rural areas. Abarca & Mora [28] showed that agricultural activities contribute significantly to water contamination with K, due to the use of fertilizers and pesticides draining into the river channels. Alternatively, given that in areas outside the city, the river flows in natural channels, a greater deposit of minerals in *P. aquaticum* may be due to natural erosion [29]. Finally, Al, Cr, Pb, and V did not vary significantly among zones of the Zamora River, but lower concentrations were found in the control. Samecka et al. [26], Cesa et al. [27], and Herrera-Núñez et al. [29] showed that the concentration of these metals in urban rivers is mainly due to the activities of textile and manufacturing industries, which are not present along the Zamora urban river. In addition, concentrations of these metals in natural waters are highly influenced by the presence of acid rain and atmospheric deposits [30], and stone extraction activities that affect deep beds [17].

Previous studies have determined that *Amblystegium riparium*, *Fontinalis antipyretica*, *Fissidens polyphyllus*, and *Rhynchostegium riparioides* are effective bioindicators of water contamination in Europe [17,21,31,32,33,34,35], due to their high bioaccumulation capacity of metals. However, only the study of Wiersma et al. [36] used aquatic *Vittia pachyloma* (Mont.) Ochyra moss for metal (e.g., Ca, Cu, Fe, Pb, Mg, and Zn) biomonitoring in the water in Chile (South America). Therefore, in our research, *Platyhypnidium aquaticum* is reported for the first time as a model species in South America. We emphasize its potential as a promising tool for the development of long-term water contamination biomonitoring programs in tropical regions, due to its efficiency and ability to bioaccumulate metals and metalloids. Nonetheless, the efficiency of *Platyhypnidium aquaticum* against tested metals is currently unknown. This is a caveat because, without knowing the bioaccumulation efficiency, our estimates of metal abundance in the environment are ultimately unknown. Furthermore, knowing the efficiency can help restoration scientists to accurately recommend what kind of metal contamination can possibly be treated with *P. aquaticum*, especially considering their relative abundance according to the study area.

In conclusion, our results point to the serious contamination of the rivers in urban Loja. The urban zones in the city of Loja have been identified as potential sources of pollution of the river because most metals, and the metalloid, are concentrated in these areas. Given the potentially high impact on the human population, solutions need to be implemented. These should include wastewater treatment systems and a prohibition to discharge pollutants in the river. The passive biomonitoring using the aquatic moss *Platyhypnidium aquaticum* provided valuable information on the state of water contamination of the Zamora River of the city of Loja, which shows that it is a reliable and economical tool to establish long-term biomonitoring in tropical areas.

## 4. Materials and Methods

### 4.1. Study Area

**Figure 2:**
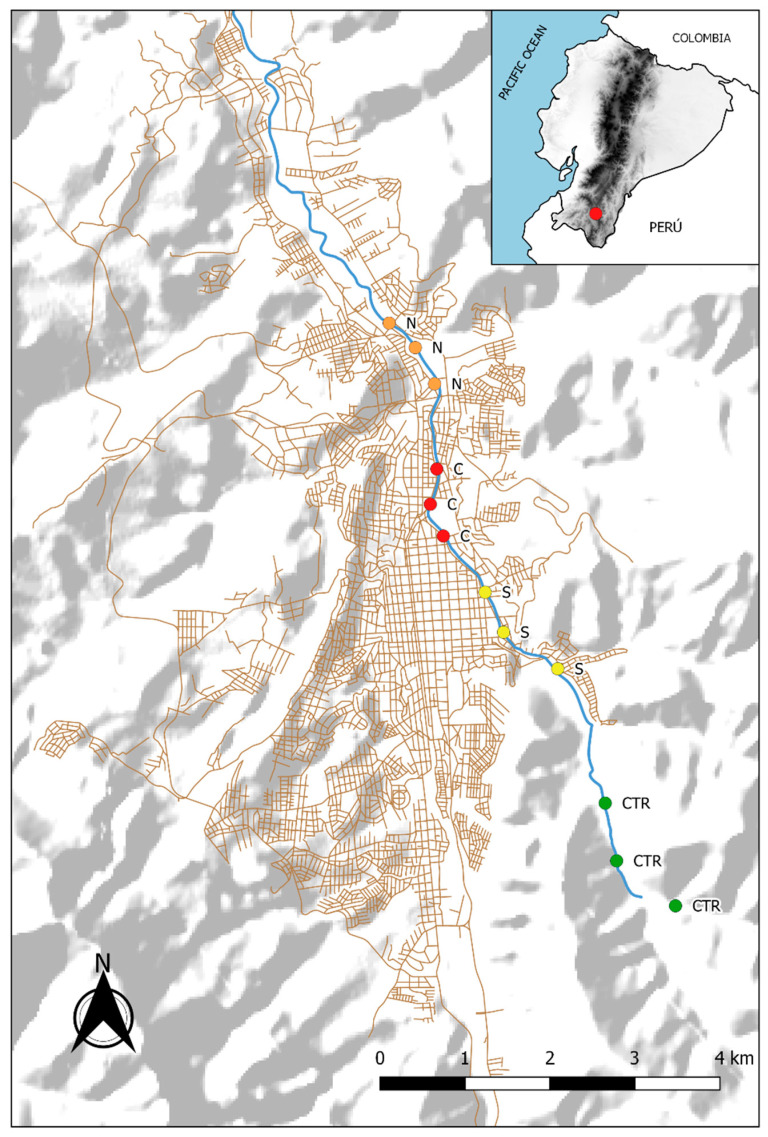
Sampling zones along Zamora River of Loja City. Control (Ctr) 1−3, South (S) 4−6, Center (C) 7−9, North (N) 10−12.

The study area was located on the riverbanks of the Zamora River, passing through Loja City (Figure 2); the area has been affected by wastewater contamination, garbage accumulation, and the extraction of stony material [7]. Field work was carried out in three urban zones (South, Center, and North) and a control zone (forest). Within each zone, we selected three independent sites. Fieldwork was conducted between January and December 2019.

The control zone (Ctr) includes the upper parts of the river basin, with low levels of water pollution and banks dominated by forest remnants. The concentrations of Al, Zn, Fe, Mn, and As in *Marchantia polymorpha* in the river reach values up to 7.82 µg g^−1^, 3.08 µg g^−1^, 0.34 µg g^−1^, 0.23 µg g^−1^, and 0 µg g^−1^, respectively [7]. The South zone (S) is characterized by a greater concentration of metals in the river and by recent urban development, and the lack of an adequate system of sewage disposal. Here, Al, Zn, Fe, Mn, and As in the river reach values up to 11.43 µg g^−1^, 11.64 µg g^−1^, 8.84 µg g^−1^, 0.77 µg g^−1^, and 0.42 µg g^−1^, respectively [7]. The Central zone (C) is characterized by a high level of water pollution with metals, a high degree of urbanization, and a large number of effluents of sewage. In this area, levels of Al, Zn, Fe, M, and As in *Marchantia polymorpha* in the river reach values up to 13.21 µg g^−1^, 13.02 µg g^−1^, 7.61 µg g^−1^, 0.54 µg g^−1^, and 4.28 µg g^−1^, respectively [7]. Finally, the North zone (N) is an urban area with high levels of metals and a high storage of microbiological load due to sewage, but the zone still has some recreational parks [8]. In this area, levels of aluminum, zinc, iron, manganese, and arsenic in *Marchantia polymorpha* in the river reach values up to 8.31 µg g^−1^, 10.71 µg g^−1^, 6.03 µg g^−1^, 0.37 µg g^−1^, and 4.4 µg g^−1^, respectively [7].

### 4.2. Platyhypnidium aquaticum Sample Processing

We chose *Platyhypnidium aquaticum* because it is an effective bioaccumulator of metals. We took five samples (0.5−1 g) of *Platyhypnidium aquaticum,* at three independent sites within three urban zones (South, Center, and North) and a forested zone (control). Therefore, concentrations of metals in *P. aquaticum* were measured in a total of 60 samples, following the protocol of Debén et al. [10]. The samples were cleaned using sieves to remove soil residues, and foreign substances were removed manually. The samples were stored in paper bags, and afterwards dried in an oven at 40 °C for 72 h. For the digestion process, 500 mg of each sample were weighed, then each sample was placed in a digestor tube and 8 mL of HNO_3_ (70%) and 2 mL of H_2_O_2_ (30%) were added, using a microwave system of high yield (Milestone SRL, Sorisole (BG), Italy), following the method of US EPA 3502. After digestion, the volume of each sample was adjusted to 100 mL with deionized water.

### 4.3. Elemental Bioccumulation in Platyhypnidium aquaticum

The concentration of 15 metals (Al, Ba, Cd, Ca, Co, Cr, Sr, Fe, Pb, Mg, Mn, K, Na, V, Zn, and a metalloid (As) was calculated based on a curve of calibration of the standards for each of the metals. The measurement was performed using two devices: the first device was a Inductively Coupled Optical Plasma Emission Spectrometer (ICP-OES) Perkin Elmer Optics 8000 (Shelton, CT, USA), for determination of the concentrations of (Co, Ba, Cr, Al, and Sr). The second device used was Atomic Absorption Spectrometry Perkin Elmer AAnalyst 400 of Perkin Elmer (Shelton, CT, USA), which allows us to obtain concentrations both in a graphite furnace for Pb, V, As, and using a flame of acetylene-air to determine the concentrations of Cd, Zn, Fe, K, Na, Ca, Mn, Mg, and Ni. All elements used here are certified, and were acquired from AccuStandard, Inc. (125 Market Street New Haven, CT 06513, USA), a company accredited to ISO Guide 34, ISO/IEC 17025, and certified to ISO 9001 [37,38].

### 4.4. Data Analysis

We evaluated the existence of differences in water contamination with 15 metals (Cd, Pb, Zn, Fe, K, Ca, Na, Mn, V, Co, Ba, Cr, Al, Sr, and Mg) and one metalloid (As) in samples of the moss *Platyhypnidium aquaticum* (A. Jaeger) M. Fleisch, from 12 localities distributed in four zones along the Zamora River in the city of Loja. We used one-way analysis of variance (ANOVA) followed by a Tukey post-hoc test whenever the assumptions of normality and homogeneity of variance were met (Shapiro Wilk and Bartlett’s test *p*-value > 0.05), i.e., for Al, Ba, Cd, Fe, Mn, Na, Zn, and As. However, when data were not normally distributed (Shapiro Wilk and Bartlett’s test, *p*-value < 0.05), we used Kruskal–Wallis one-way ANOVA on ranks, followed by Dunn’s pairwise comparison, i.e., for Ca, Cr, Co, Pb, Mg, K, Sr, and V. Analyses were done in R 3.2.2 [39] using the “dunn.test” package [40].

## Figures and Tables

**Table 1:** Results of the one-way ANOVA and Kruskal-Wallis on metal concentrations in *Platyhypnidium aquaticum* (µg g^−1^) in the Zamora River of Loja City. The median and standard error (SD) of the concentrations of the metals and the metalloid are shown.

One-Way ANOVA										
Metals	Control		South		Center		North		Anova	
	Median	SD	Median	SD	Median	SD	Median	SD	F-Valor	*p*-Valor
Aluminum	1355.12	343.11	1390.17	671.93	1505.45	407.69	1370.42	516.18	0.26	0.853
Arsenic	6.81	3.59	22.2	5.19	17.25	5.66	21.95	8.19	20.89	<0.001
Barium	15.3	2.99	50.29	18.52	26.48	8.96	21.94	6.11	31.84	<0.001
Cadmium	0.72	0.75	4.92	1.23	5.25	0.66	6.64	0.81	134.2	<0.001
Iron	510.13	164.04	1179.52	301.68	977.03	320.57	1076.6	197.3	18.85	<0.001
Manganese	0.4	1.02	2412.21	1301.38	936.49	1032.78	536.62	549.81	78.87	<0.001
Sodium	50.89	6.12	72.38	26.42	90.04	25.34	96.06	20.66	12.78	<0.001
Zinc	185.08	138.38	913.87	256.44	640.4	245.75	559.55	107.93	17.73	<0.001
**Kruskal-Wallis Anova**										
Calcium	12.65	3.79	18.56	14.53	18.4	16.28	12.07	6.9	0.68	0.878
Chromium	0.7	0.35	2.14	3.8	4.57	11.94	0.79	0.41	6.6	0.086
Cobalt	0	0	26.06	20.64	6.99	8.06	1.81	2.57	22.9	<0.001
Lead	10.62	6.97	10.39	8.86	12.86	10.68	12.74	9.68	3.6	0.308
Magnesium	53.1	9.54	74.35	9.3	59.79	22.71	60.37	6.89	21.26	0.001
Potassium	127.64	21.06	60.92	41.76	120.75	145.4	111.93	63.67	12.25	0.006
Strontium	8.99	1.72	10.07	1.94	7.85	1.31	7.12	1.54	16	0.001
Vanadium	27.69	11.42	38.24	13.42	37.55	8.51	32.86	11.01	5.46	0.141
